# The Prevention of Implant Surface Alterations in the Treatment of Peri-Implantitis: Comparison of Three Different Mechanical and Physical Treatments

**DOI:** 10.3390/ijerph17082624

**Published:** 2020-04-11

**Authors:** Marco Lollobrigida, Lorenzo Fortunato, Giorgio Serafini, Giulia Mazzucchi, Giuseppina Bozzuto, Agnese Molinari, Emanuele Serra, Francesca Menchini, Iole Vozza, Alberto De Biase

**Affiliations:** 1 Department of Oral and Maxillofacial Sciences, Sapienza University of Rome, 00161 Rome, Italy; 2 National Centre of Drug Research and Evaluation, Istituto Superiore di Sanità, 00161 Rome, Italyagnese.molinari@iss.it (A.M.); 3 Sustainability Department, ENEA, Casaccia Research Center, 00123 Rome, Italy; 4 Energy Technology Department, ENEA, Casaccia Research Center, 00123 Rome, Italy

**Keywords:** peri-implantitis, implant decontamination, peri-implantitis treatment, peri-implantitis therapy, mechanical treatments, dental implants

## Abstract

The surgical treatment of peri-implantitis is currently based on the removal of biofilms from the implant surface by primary means of mechanical and physical treatments. However, such approaches often determine some alterations of the implant surface with detrimental effects on re-osseointegration. This study aims to evaluate the effects of four different mechanical and physical treatments on titanium samples with moderately rough surface. Air powder abrasion (AP) with glycine powder, a titanium brush (TB) and a diode laser at 3 W (L3) and 4 W (L4) were tested. Surface morphology, roughness and chemical composition were then assessed by scanning electron microscope (SEM), white light interferometer and X-ray photoelectron spectroscopy (XPS), respectively. The microscopic analysis revealed significant alterations in surface morphology on TB samples, while AP and L3 had only a minor or null impact. L4 samples revealed signs of overheating due to the excessive power. Nevertheless, the overall roughness of the samples was not significantly altered in terms of roughness parameters. Similarly, surface chemical composition was not significantly affected by the treatments. Among the treatments tested in this study, air powder abrasion with glycine powder and 3 W diode laser had the lowest impact on surface physicochemical properties.

## 1. Introduction

Peri-implantitis is a pathological condition that affects tissues around dental implants, characterized by inflammation in the peri-implant mucosa and progressive bone loss [1]. Peri-implantitis has become a relatively common disease in dentistry, with an estimated subject-based prevalence between 18.8% and 34% [2,3,4], though higher prevalence rates, based on different case definitions, have been also reported [5]. The increased prevalence may be attributable to the greater number of subjects treated with implants than in the past but may also depend on major diagnostic accuracy and attention to monitoring the peri-implant tissues.

Most literature studies identify bacterial biofilms as the primary etiological factor for the initiation and progression of peri-implantitis [6,7]. According to some authors, other factors may initiate an aseptic bone resorption, followed in a later stage by bacterial colonization [8]. However, once peri-implantitis has been established, the cardinal aim of therapy consists in the disruption of the biofilm adhered to implant surface to resolve the inflammation. This entails two major difficulties: firstly, the need for a complete surgical decontamination of the implant, dislodging structured biofilms, as well as the removal of bacterial and matrix remnants from surfaces that are highly retentive; secondly, the need to prevent surface alterations during the decontamination procedures, preserving both the physical and chemical properties of the implant and obtaining a re-osseointegration of the previously contaminated surface [9]. 

On the one hand, several mechanical and chemical methods have been described for decontaminating implants; however, less is known about their impact on the implant surface. Based on the nature of the treatment, possible alterations include changes of both surface morphology and chemical composition [10,11,12]. It is well established that osteoconduction strictly depends on surface characteristics that enhance early platelet activation and cytokine release and that improve the anchorage of the provisional fibrin matrix [13,14]. Noncontrolled surface alterations due to implant decontamination could then prevent re-osseointegration and impair long term results.

In this regard, this in vitro study aims to evaluate and compare the morphological, roughness and chemical alterations of three decontaminating treatments commonly used in the surgical treatment of peri-implantitis.

## 2. Materials and Methods

Sample disks of commercially pure titanium grade 4 ASTM (American Society for Testing and Materials, West Conshohocken, Pennsylvania, US) were used in this study. The disks (10 mm diameter, 1.5 mm thickness) were moderately rough (S_a_ (average roughness) 1.30 µm) with SLA (sandblasted and acid-etched) surface (Camlog Promote, Basel, Switzerland). Each disk was sterilized by autoclaving before each experimental procedure.

**Figure 1:**
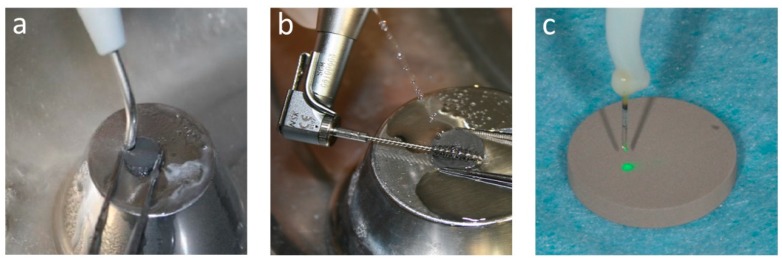
(**a**) Air powder abrasion with glycine powder; (**b**) titanium brush; (**c**) diode laser.

The following mechanical and physical treatments were tested:

Air powder abrasion (AP): three disks were treated by air polishing (EasyjetPerio, Mectron Medical Technologies, Carasco, Italy) with glycine powder (Mectron Glycine Powder, Mectron Medical Technologies, Carasco, Italy). The duration of the treatment was 40s with an angle of incidence of 90° and a ~2 mm distance between the tip and the surface, moving the instrument over the entire surface (Figure 1a). At the end of the treatment, the samples were washed with 30 cc of distilled water for 60 s to remove the residual powder.

Titanium brush (TB): three disks were treated using a titanium brush (TiBrush, Straumann, Basel, Switzerland) in continuous rotation at 600 rpm for 40s (Figure 1b). The treatment was carried out under continuous irrigation with 20 cc of distilled water with the long axis of the brush parallel to the surface. At the end of the treatment all the disks were irrigated with 30 cc of distilled water for 60s.

Laser: a total of six disks were treated by a diode laser (FOX Laser Diode, A.R.C. Laser GmbH, Nürnberg, Germany) with optical fiber of 810 nm wavelength. Three disks were irradiated at 3 W of power (L3) and other three disks at 4 W (L4). Each sample was treated for 40s, without cooling, following a sinusoidal route to cover the whole surface (Figure 1c). The tip was oriented perpendicular to the disk at the minimal noncontact distance (~0.5–1 mm) from the surface. 

Control disks (CTR) were analyzed as provided by the manufacturer.

### 2.1. Scanning Electron Microscopy (SEM) Analysis

Specimens were analyzed using a field emission gun scanning electron microscope (FEG-SEM) (Inspect FTM, FEI Company, Hillsboro, OR, USA). The samples were observed with a gold coating of 30 nm to allow a better characterization. Each surface was observed at different magnifications with a potential difference of 10 kV.

### 2.2. Topographical Analysis

Surface microtopography was analyzed using a white light interferometer (NewView 5000TM, Zygo, Middlefield, USA). Measurements were performed on three areas of 0.71 × 0.53 mm, randomly selected on each sample. No filter was applied. Data were processed using MetroPro software (Zygo, Middlefield, USA). As suggested by the guidelines of Wennerberg and Albrektsson [15], the following amplitude, space and hybrid parameters were evaluated:

S_a_, arithmetic average roughness: arithmetic mean of the distances between the points of a surface (S_a_) or of a profile (R_a_) and an average reference plane;

S_q_, average quadratic roughness: quadratic mean of the distances between points of a surface (S_a_) or of a profile (R_a_) and an average reference plane. This parameter, compared to the previous one, highlights the existence of values that deviate greatly from the central values; 

S_cx_ (summit spacing): average spacing between peaks, calculated as the square root of the measurement area divided by peaks’ number.

### 2.3. Spectroscopic Analysis

Surface chemical analysis was carried out using X-ray photoelectron spectroscopy (XPS) (Escalab MKII, Vacuum Generators). Before treatment, each sample, previously sterilized by autoclaving, was given an ultrasonic bath with acetone and then with 95% ethyl alcohol, each for 2 min, and air-dried. 

## 3. Results

### 3.1. SEM Analysis 

**Figure 2:**
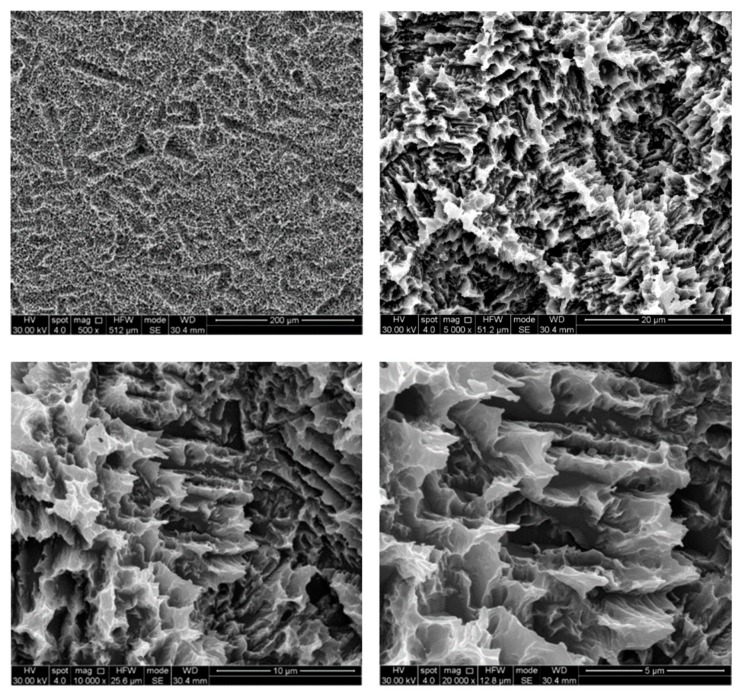
SEM images of control disk (CTR) samples. Magnifications from 500× to 20,000×.

**Figure 3:**
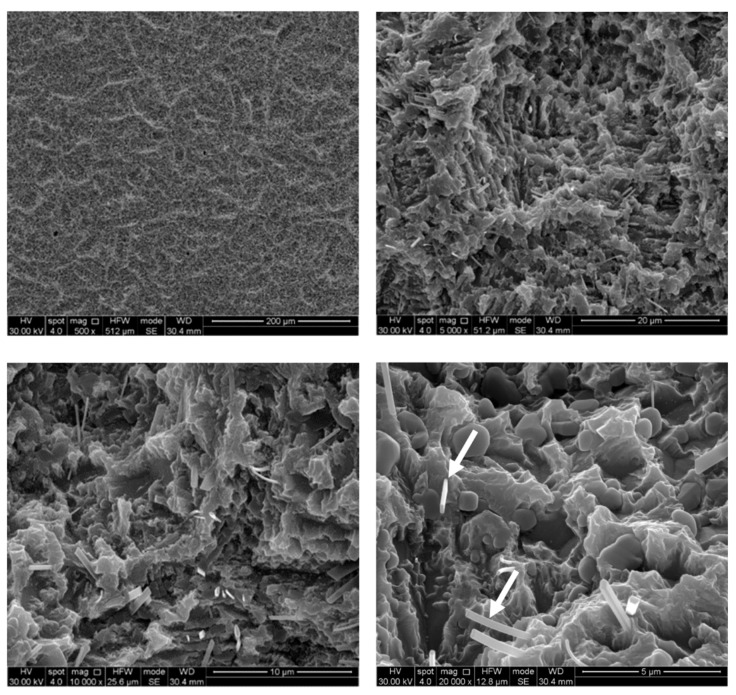
SEM images of air powder abrasion (AP) samples. Glycine particle residues (white arrows) and rounded edges can be observed. Magnifications from 500× to 20,000×.

**Figure 4:**
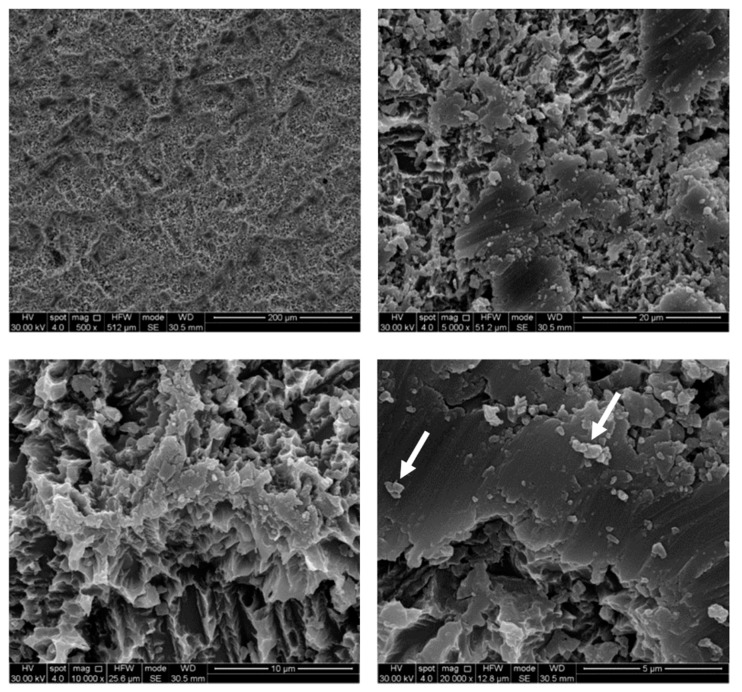
SEM images of titanium brush (TB) samples. Smoothed portions and titanium particles (white arrows) are clearly visible at high magnifications. Magnifications from 500× to 20,000×.

**Figure 5:**
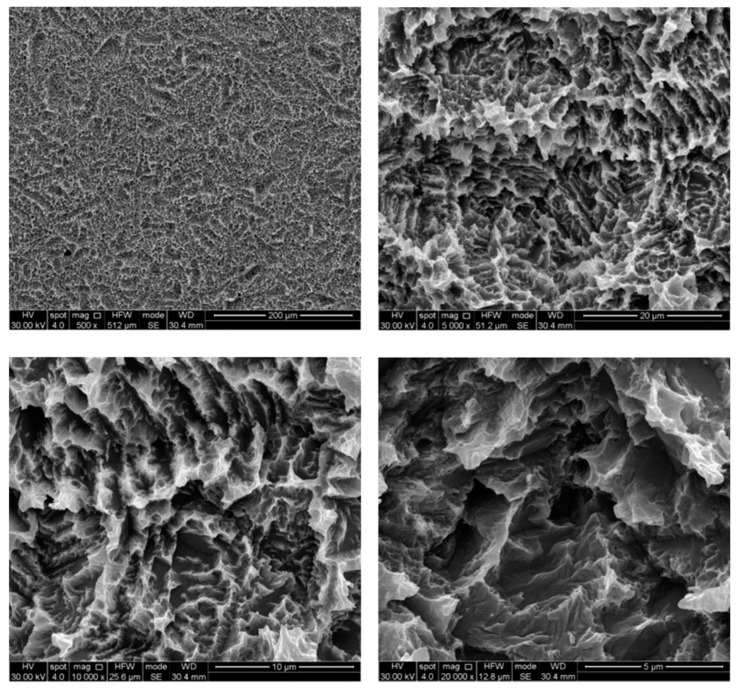
SEM images of diode laser at 3 W (L3) samples. No signs of alteration are visible. Magnifications from 500× to 20,000×.

**Figure 6:**
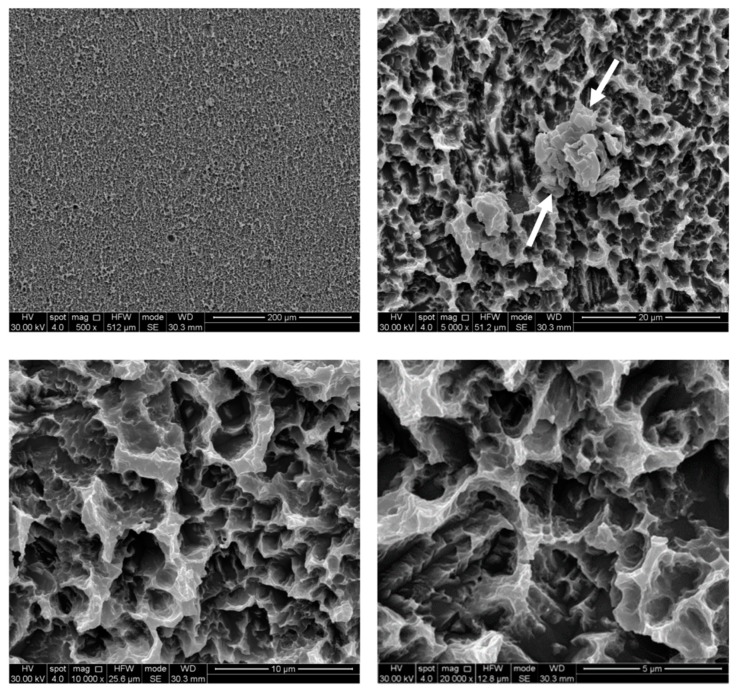
SEM images of diode laser at 4 W (L4) samples. Laser spot melting (white arrows) could be visible at 5000×. Magnifications from 500× to 20,000×.

The untreated control disks (CTR) were observed as furnished by the manufacturer, revealing the typical microporous structure of SLA surfaces obtained through a combined sandblasting and etching process (Figure 2). The surface morphology of these samples was irregular, with ridges and sharp edges. At low magnification, disks treated by AP did not show substantial differences compared to the CTR samples, while residues of glycine powder could be detected at higher magnifications (Figure 3). Slight alterations in surface morphology were also appreciable, consisting of a slight rounding of the surface edges. TB samples were characterized by macroscopic alterations, like coarse scratches (Figure 4). At 5000× magnification these alterations were characterized by smoothed portions of titanium. Further magnifications also revealed the presence of titanium particles. As for laser-irradiated disks, L3 samples did not show any alteration of the surface microtexture (Figure 5), while signs of localized melting due to temperature rise could be detected on L4 samples (Figure 6).

### 3.2. Topographical Analysis

The results of the roughness analysis are shown in Table 1.

As for the average roughness (S_a_), the order of roughness, from the smoothest to the roughest surface, is L4 < AP < L3 < CTR < TB. Similar values of parameters were observed for CTR and L3 samples. No significant differences could be highlighted in surface roughness, on the whole.

### 3.3. Spectroscopic Analysis

**Figure 7:**
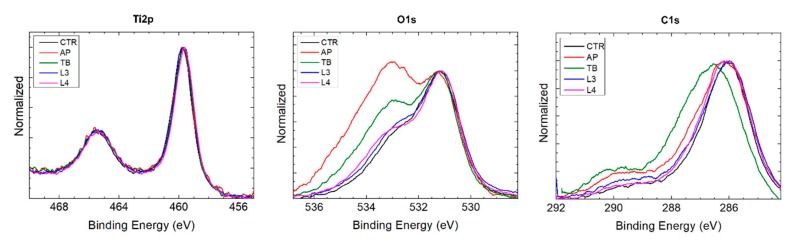
XPS (X-ray photoelectron spectroscopy) analysis of samples showing the spectra of Ti2p, O1s and C1s.

All spectra showed very intense peaks of carbon, oxygen and titanium (Figure 7). In addition to these elements, the presence of nitrogen was also revealed on all samples, although lower than 3%.

Titanium was found to be present almost exclusively in bonded form with two distinct peaks corresponding to Ti2p1/2 and Ti2p3/2. The binding energy of Ti2p3/2 at about 459.7 eV had a chemical shift of about 4.3 eV compared to metallic titanium and a spin orbit splitting ΔE (2p3/2–2p1/2) = 5.6 eV typical of TiO_2_ for all samples, including CTR. In all samples, the O1s spectrum showed several overlying peaks, attributable to different oxygen compounds. The lower energy peak corresponds to the TiO_2_ bond, partially superimposed on that of adsorbed oxygen. The peaks at higher energies, particularly evident in AP and TB samples, correspond to hydroxides, carbonates and other compounds. In all samples, the C1s spectrum also showed several overlapping peaks, attributable to different carbon compounds. The lower energy peak corresponds to adsorbed atomic carbon, while at higher energies the peaks correspond to the C–H, C–O, C=O and other bonds.

By analyzing the spectra of the individual elements, it was possible to quantify the atomic percentage composition of the species present on the surface of the samples (Table 2). However, given the intrinsic error associated with this technique, which is of 10%–15%, the obtained values must be considered as indicative.

## 4. Discussion

In this study, four different mechanical and physical decontamination treatments were tested on titanium samples with SLA surface to assess the effects on surface morphology, topography and chemical composition. 

Upon microscopic analysis, almost all the samples presented some alterations. The morphological changes were closely related to the type and nature of the treatments and consisted of rounded edges, smoothening and/or detachments of titanium portions and spot melting. Particles of titanium and glycine were also detected on AP and TB samples. The results obtained with air powder abrasion agree with the observations of Ramaglia et al. [16] and John et al. [17], who reported minimal surface alterations with air polishing in standard operating conditions. Glycine powder is then confirmed to be a relatively noninvasive blasting medium, while major alterations were reported with bicarbonate powder [18,19]. Schwarz et al. [18] reported a significant impact of sodium bicarbonate on titanium surface compared to glycine powder. These findings were also confirmed by Tastepe [20] by comparing different types of powders. The permanence of residual granules was also observed in these studies [18,19,20] and appears to be an inevitable consequence of abrasive treatments. Notwithstanding, Tastepe et al. [20] reported a higher biocompatibility of titanium surfaces treated with osteoconductive powders compared to nontreated samples. Though it has not been fully elucidated whether residual particles can negatively affect bone formation, the use of biocompatible and osteoconductive powders may thus represent a cautious or even useful solution [21]. Significant alterations of surface morphology were observed on the samples treated with the titanium brush, confirming the findings of previous reports [22,23]. Though more effective than ultrasonic devices, titanium brushes were demonstrated to provoke significant damage on titanium implants [23]. In our study, this resulted in a flattening of the surface ridges and in the formation of titanium debris. Moreover, since only the most prominent portions of the surface were damaged by the brush, this suggests that the lower portions may remain contaminated. As for diode laser, the results of this study are partially in agreement with the literature, since other authors reported no significant alterations on titanium samples irradiated with this type of laser [24,25], while we observed spot melting using 4 W power. This can be related to temperature increase due to the longer duration of the treatment (40 s) on relatively small disks, to the continuous irradiation mode without cooling and to the higher power compared to other studies. However, our findings are still in contrast with the results of Castro [26], who tested the diode laser at 15 W without significant surface damage. This can depend, however, on the treatment time adopted in that report (60 s) for the entire surface of the implant.

Despite the alterations described above, the micro-topographical analysis did not reveal significant differences in roughness parameters between the test samples and the untreated control. Among the treatments, the 3 W diode laser resulted in minor changes, as already found by other authors [27,28]. The slight decrease of S_a_ values observed with air powder abrasion has also been reported in other studies [29] and may be explained with the rounding of the surface crests. Similarly, the slight increase of S_a_ on the samples treated with the titanium brush can be related to the formation of metal debris. However, none of the tested treatments determined significant variations in surface roughness. 

Finally, the results of the XPS analysis revealed a minimal impact of the treatments on the chemical composition of the samples. In fact, a comparable atomic composition was found among the test and control samples. In particular, the percentages of titanium were lower than expected (4%–11%), while higher percentages of carbon (36%–50%) and oxygen (43%–50%) were detected, similarly to what was reported by Al-Hashedi et al. [30]. Contrariwise, Tastepe et al. [20] found significantly higher amounts of titanium after different decontamination treatments. This can be explained by the different analysis techniques, since energy-dispersive X-ray spectrometry (EDS) analyzes the bulk composition of materials, while XPS analyzes the most superficial atomic layers. Despite the intrinsic error of 10%–15%, the scarcity of titanium is evident in our study. This can be related to the chemical nature of titanium that, being a getter element, attracts and absorbs atoms and molecules of various nature. The presence of large quantities of carbon and oxygen is however also a consequence of the decontamination treatments and exposure to atmospheric air, while nitrogen, usually found in case of bacterial contamination, in this case is attributable exclusively to air contact. Finally, the higher percentages of carbon found on AP samples are attributable to the residues of glycine powder deposited above the surface, confirming the microscopic observations.

## 5. Conclusions

The present study has some limitations. First, the decontamination efficacy of the tested treatments could not be assessed due to the absence of bacterial contamination. Second, we have not assessed surface biocompatibility and osteoconduction after the treatments, which will be the object of future research. Within these limitations, the results of this study indicate, overall, a limited impact of the decontamination treatments on the morphology, microtopography and surface chemical composition of titanium samples with SLA surface. More specifically, air powder abrasion with glycine powder and 3 W diode laser were found to be less invasive than titanium brush and 4 W laser. Future clinical studies should then elucidate whether the failure in treating peri-implantitis is more likely related to surface alterations due to long-term bacterial colonization, invasive decontamination procedures or residual bacterial contamination.

## Figures and Tables

**Table 1:** Surface roughness analysis.

	CTR	AP	TB	L3	L4
S_a_ (µm)	2.938 ± 0.05	2.584 ± 0.21	3.197 ± 0.32	2.894 ± 0.07	2.434 ± 0.10
S_q_ (µm)	4.353 ± 0.53	3.579 ± 0.33	4.303 ± 0.45	4.235 ± 0.23	3.727 ± 0.41
S_cx_ (µm)	21.661 ± 1.89	20.760 ± 0.56	20.615 ± 0.60	21.944 ± 0.92	20.615 ± 0.44

S_a_ and S_q_ = average surface height deviation amplitude and root-mean-square roughness; S_cx_ = average spacing between the peaks. CTR, control. AP, air powder abrasion. TB, titanium brush. L3, diode laser at 3 W. L4, diode laser at 4 W.

**Table 2:** Atomic percentage composition of samples.

	Titanium (%)	Oxygen (%)	Carbon (%)	Sodium (%)
CTR	9	43	45	3
AP	4	43	50	3
TB	8	48	42	2
L3	11	50	36	3
